# Predictive role of ferroptosis-related long non-coding RNAs in bladder cancer and their association with immune microenvironment and immunotherapy response

**DOI:** 10.1186/s12957-022-02514-4

**Published:** 2022-02-24

**Authors:** Jingchao Liu, Zhipeng Zhang, Xiaodong Liu, Wei Zhang, Lingfeng Meng, Jiawen Wang, Zhengtong Lv, Haoran Xia, Yaoguang Zhang, Jianye Wang

**Affiliations:** 1 Department of Urology, Beijing Hospital, National Center of Gerontology, Institute of Geriatric Medicine, Chinese Academy of Medical Sciences, No. 1 DaHua Road, Dong Dan, Beijing, 100730 China; 2 Graduate School of Peking Union Medical College, Chinese Academy of Medical Sciences, 9 DongDan Santiao, Beijing, 100730 China

**Keywords:** Ferroptosis, lncRNA, Bladder cancer, Immunotherapy, Immune microenvironment

## Abstract

**Background:**

We have previously reported that ferroptosis has an important role in bladder cancer development. In this study, we aimed to further explore the possible predictive ability of ferroptosis-related long non-coding RNAs (lncRNAs) in bladder cancer and their relation with immune microenvironment and immunotherapy response.

**Materials and methods:**

The ferroptosis-related lncRNAs were identified by Pearson’s correlation analysis. The predictive lncRNA signature was developed by univariate and multivariate regression analyses. Only the main effects of independent variables in multivariate analysis were included in this signature. The TCGA dataset was defined as the training cohort and GEO was the validation cohort in this study. All samples were grouped into a high- or low-risk group depending on risk signature. The prognostic role of lncRNA signature was explored through survival analysis and receiver operating characteristic curve (ROC) analysis in both TCGA and GEO cohorts. Additionally, the independent prognostic ability of the lncRNA signature was confirmed by multivariate independent analysis. Furthermore, the relationship between lncRNAs and immune microenvironment as well as immunotherapy response in bladder cancers was studied.

**Results:**

The Kaplan–Meier curves identified significantly poorer overall survival outcomes for high-risk groups in both TCGA (*p* < 0.001) and GEO (*p* < 0.001) cohorts. The area under the curve (AUC) during ROC analysis of 1, 3, and 5 years was 0.781 ± 0.046, 0.784 ± 0.027, and 0.817 ± 0.025, respectively, in the TCGA cohort and 0.665 ± 0.177, 0.719 ± 0.068, and 0.791 ± 0.055, respectively, in the GEO cohort. The multivariate independent analysis in TCGA cohort identified age (*p* = 0.003), stage (*p* < 0.001), and signature risk score (*p* < 0.001) as independent risk factors for overall survival. Furthermore, this study demonstrated a significant difference in infiltration levels of various immune cells between high- and low-risk groups. The high risk group tended to have a lower expression of proteins including PD1 (*p* < 0.01), PD-L1 (*p* < 0.01), CTLA-4 (*p* < 0.05), etc. corresponding to various immune checkpoints. Additionally, the immunotherapy trial confirmed that the high-risk group tended to have a poorer treatment response than the low-risk group (*p* < 0.001).

**Conclusions:**

The ferroptosis-related lncRNAs exhibited a good predictive capacity for overall survival in bladder cancer. Additionally, they could be utilized to reveal tumour-immune microenvironment and immunotherapy responses.

**Supplementary Information:**

The online version contains supplementary material available at 10.1186/s12957-022-02514-4.

## Background

Bladder cancer was reported to cause more than 200,000 annual deaths worldwide, and the treatment for bladder cancer was not significantly changed during the past decades [1]. Larger than 50% of bladder cancer patients were classified as non-muscle-invasive cancer (NMIBC) and about 30% of patients were confirmed as muscle-invasive bladder cancer (MIBC) [2]. More than one-third of NMIBC could develop into MIBC or distant metastasis and further lead to great health and economic threat for the society [3]. During the past decades, the stage, grade, or various characteristic mRNAs of bladder cancer was perceived as a vital tool to predict survival and to plan therapeutic strategies in clinical practice [4–6]. Transurethral resection or radical cystectomy was currently suggested as effective surgical operations for bladder cancer [7]. Cisplatin-based chemotherapy has been suggested as the first-line strategy for advanced or metastatic bladder cancer and immunotherapy was also suggested as the second-line treatment [8]. On one hand, current treatment responses for chemotherapy or immunotherapy were rather limited and highly heterogeneous. On the other hand, the patients posing the same stage or grade in clinical often obtained different long-term prognoses after receiving totally similar treatment. So, there must be other important mechanisms underlying the bladder development which was important for improving bladder cancer prognosis or biomarker in future.

We have previously reported that ferroptosis has an important role in bladder cancer development, and ferroptosis has become a novel research topic for its targeted therapy [9]. The use of ferroptosis inducers had been identified as a potential therapeutic method to induce cell death in malignant cancers which were resistant to current traditional treatments [10]. Ferroptosis was reported as a unique cell death procedure which was totally different from previous known programmed cell death including apoptosis and autophagy and it mainly manifested as iron-dependent and reactive oxygen species accumulation in biologic cells [11, 12]. The resistance to ferroptosis could significantly affect the efficacy of sorafenib and lead to poor survival prognosis according to recent researches [13]. Ferroptosis-related mRNAs had also been reported to be responsible for predicting survival outcomes in colon cancers [14]. However, studies concerning the roles of ferroptosis-related LncRNAs in malignancies were rather limited. LncRNA was defined as RNA having > 200 nucleotides that did not contain any code for proteins in the human body but exhibited vital roles during various development of tumours [15]. Several studies have reported that lncRNAs exhibit a close relationship with the ferroptosis-related process in various malignant tumours [16–18]. Various studies have reported that lncRNAs participate in various biological activities including metabolism, infection, and immune response. All these activities have been reported to have a close relationship with the ferroptosis mechanism. Wang et al. recently reported that lncRNA LINC00336, as a competing endogenous RNA, was responsible for lung cancer development through inhibiting ferroptosis [19]. The dysregulation of LINC00618 could promote vincristine-induced ferroptosis in human leukaemia and could be further useful in improving prognosis [20]. Additionally, the lncRNA PVT1 was reported to affect ferroptosis through miR-214 and further regulate the expression of TFR1 and TP53 [21].

Our previous study was the first to report an important role of ferroptosis-related protein-coding genes in bladder cancer. Several studies have confirmed the regulating roles of ferroptosis in the development of bladder cancer [9, 22, 23]. Depending on recent cutting-edge studies concerning the relationship between ferroptosis and lncRNAs, this study aimed to explore the possible predictive ability of ferroptosis-related lncRNAs and their possible relation with immune microenvironment and immunotherapy response in bladder cancer.

## Materials and methods

### Data collection and processing

RNA-sequencing data of 430 bladder samples (19 normal samples and 411 bladder tumours) were directly downloaded from The Cancer Genome Atlas (TCGA) database in the format of FPKM, which were cross-referenced in Supplementary Table 1. (https://portal.gdc.cancer.gov/) The clinical information files of 409 bladder tumours were also downloaded from TCGA cohort. (Supplementary Table 2) The mRNA data and corresponding clinical files of 256 bladder samples were also derived from the GEO database (GSE13507). (Supplementary Table 3) (https://www.ncbi.nlm.nih.gov) Only 165 bladder tumours posed available clinical information in the GEO cohort. (Supplementary Table 4) All downloaded expression files were normalized utilizing “limma” packages (Ritchie, M.E., 2015) in R software (R version 4.0.3). All included samples data were derived from public databases and no ethical approval was needed in the study. All data proceeding and downloading in analysis strictly followed the guidelines of the TCGA and GEO databases. The detailed mRNA expression data and lncRNA expression data of TCGA and GEO samples were further classified depending on the annotation file of Genome Reference Consortium Human Build of GENCODE in Supplementary Table 5 [24]. The whole ferroptosis-related encoding genes were derived from FerrDb [25], a novel database which was dedicated to collecting and collating the latest relevant research on ferroptosis-related regulators. Samples lacking relative clinical information were excluded from further analysis in this study. The lncRNA expression (Supplementary Table 6) and mRNA expression levels (Supplementary Table 7) of TCGA cohorts were separately distinguished out depending on the above methods. The lncRNA expression and mRNA expression levels of GEO cohorts were also identified with the same methods. (Supplementary Tables 8 and 9)

**Table 1:** AUCs of ROC analysis concerning predictive roles of various parameters for overall survival

	1 year	2 years	3 years	4 years	5 years
**TCGA cohort**					
lncRNA risk	0.781 ± 0.046	0.789 ± 0.030	0.784 ± 0.027	0.797±0.026	0.817 ± 0.025
Age	0.636 ± 0.056	0.563 ± 0.035	0.591 ± 0.032	0.579±0.031	0.588 ± 0.030
Gender	0.479 ± 0.056	0.482 ± 0.036	0.485 ± 0.032	0.482±0.032	0.500 ± 0.031
Grade	0.531 ± 0.057	0.528 ± 0.036	0.466 ± 0.032	0.468±0.031	0.469 ± 0.031
Stage	0.597 ± 0.056	0.622 ± 0.034	0.610 ± 0.032	0.621±0.031	0.641 ± 0.030
**GEO cohort**					
lncRNA risk	0.665 ± 0.177	0.720 ± 0.086	0.719 ± 0.068	0.750±0.060	0.791 ± 0.055
Age	0.659 ± 0.140	0.649 ± 0.079	0.665 ± 0.066	0.671±0.057	0.701 ± 0.052
Gender	0.429 ± 0.139	0.433 ± 0.081	0.450 ± 0.066	0.453±0.059	0.447 ± 0.055
Grade	0.695 ± 0.147	0.657 ± 0.078	0.685 ± 0.064	0.676±0.058	0.662 ± 0.053

### Identification of ferroptosis-related LncRNAs in bladder cancer

The ferroptosis-related genes were derived from the FerrDb database, and their detailed gene expression files were identified from TCGA and GEO expression documents. Further, Pearson’s correlation analysis was conducted between ferroptosis-related genes and total lncRNA expression data. The ferroptosis-related lncRNAs were finally identified when defining the |correlation coefficient| > 0.4 and *p* < 0.001. The differentially expressed ferroptosis-related genes (DEGs) between bladder cancer and normal tissues were also explored defining false discovery rate (FDR) < 0.05 and |log_2_FC|≥1. (FC: fold change) Further functional analysis including Gene Ontology (GO) and Kyoto Encyclopedia of Genes and Genomes (KEGG) analysis were performed using the “ggplot2” package (H. Wickham., 2016) with R software.

### Construction and evaluation of ferroptosis LncRNA signature of prognostic value for bladder cancer

On the basis of ferroptosis lncRNA expression levels and corresponding clinical information, univariate Cox regression analysis was firstly performed to identify the prognostic ferroptosis-related lncRNAs for bladder cancer defining the *p*-value < 0.001. Then, the prognostic ferroptosis-related lncRNAs were included in the further multivariate Cox regression analysis to evaluate each prognostic contribution for patients’ overall survival. Depending on multivariate analysis results, the optimal ferroptosis lncRNA signature was constructed with the lowest Akaike information criterion value. The detailed risk score formula was as follows: The riskScore = *e*^sum (ferroptosis lncRNA expression * corresponding regression coefficient)^. All TCGA samples were then grouped into high- or low-risk groups according to the median risk score in the whole TCGA cohort. The ferroptosis-related lncRNA signature was also calculated in GEO cohorts. The median risk score of the whole GEO cohorts was also used to group patients into a high- or low-risk group.

### Kaplan-Meier analysis, ROC analysis, and decision curve analysis

To test the prognostic value of this ferroptosis lncRNA signature, Kaplan–Meier analysis with the log-rank test was utilized to compare the differences of overall survivals between different risk groups for both TCGA and GEO cohorts. For the TCGA cohort, the ferroptosis lncRNA risk signature combining various clinical data including age, gender, tumour grade, and stage was used to perform univariate and multivariate logistic regression analysis to identify the independent risk factors for bladder cancer overall survival. For the GEO cohort, the ferroptosis risk score and various clinical data such as age, gender, and tumour grade were included in the univariate and multivariate logistic regression analysis to explore the independent prognostic value of the risk signature. The “survival” package (Therneau T, 2021) and function formula for the Cox proportional hazards model were utilized to conduct the above Cox regression analysis. Then, to compare the predictive accuracy of ferroptosis lncRNA signature with other clinical information, “survivalROC” packages (Therneau T, 2021) in R were utilized to perform time-dependent ROC analysis. “DCA” and “survival” packages (Therneau T, 2021) in R software were also used to perform decision curve analysis (DCA) to compare the prognostic value of various clinical data.

### Correlation analysis between clinical data and high or low-risk groups and co-expression network illustration

To investigate the correlation between the risk groups and various clinical data including age, sex, stage, grade, the “limma” (Ritchie, M.E., 2015) and “heatmap” packages (Raivo Kolde, 2019) in R software were used in this study. All significant clinical parameters were marked with an asterisk in the upper right corner using a heatmap. To illustrate the correlation between target ferroptosis-related lncRNAs and corresponding ferroptosis-related mRNAs, we developed a co-expression network using Cytoscape (http://www.cytoscape.org/). The previous co-expression analysis results were input into Cytoscape at this stage.

### Gene-set enrichment analysis (GSEA) for high- and low-risk groups

To further explore the possible biological procedures correlated with the lncRNA signatures, the gene-set enrichment analysis (GSEA) (http://www.broadinstitute.org/gsea) analysis was also conducted. The top 6 significantly enriched biological functions in high- or low-risk groups were illustrated in this study (*p* < 0.05).

### Evaluation for tumour-infiltrating immune cells between high- and low-risk groups

To explore the difference of tumour-infiltrating immune cells between high- and low-risk groups, the infiltrating levels of different estimating algorithms including CIBERSORT [26], QUANTISEQ [27], TIMER [28], XCELL [29], Microenvironment Cell [30], and Estimating the Proportion of Immune and Cancer cells (EPIC) [31] were used to analyse the immune-infiltration difference among ferroptosis lncRNA signatures. Furthermore, the different expression levels of immune checkpoint-related genes as well as m6A-related genes among different lncRNA risk groups were also explored by the “ggplot2”, (H.Wickham.,2016) “ggpubr” (Alboukadel Kassambara, 2020), and “limma” (Ritchie, M.E., 2015) packages in R software. The predictive value of the lncRNA signature for immunotherapy responses was also explored during this study.

### Statistical analysis

Student’s *t*-test was utilized to explore DEGs between cancer and noncancer tissues. The categorical variables were compared using the chi-squared test. The relative differences among 3 or more groups were analysed by one-way ANOVA. The Kaplan–Meier analysis was used to explore the survival difference between high and low ferroptosis lncRNA risk groups. R software (version 4.0.3) or SPSS (version 25.0) was used to perform statistic analysis during the current research. A *p*-value < 0.05 of a two-tail should be present to be significant.

## Results

### Identification of ferroptosis-related lncRNAs and construction of ferroptosis-related LncRNA signature

**Fig. 1:**
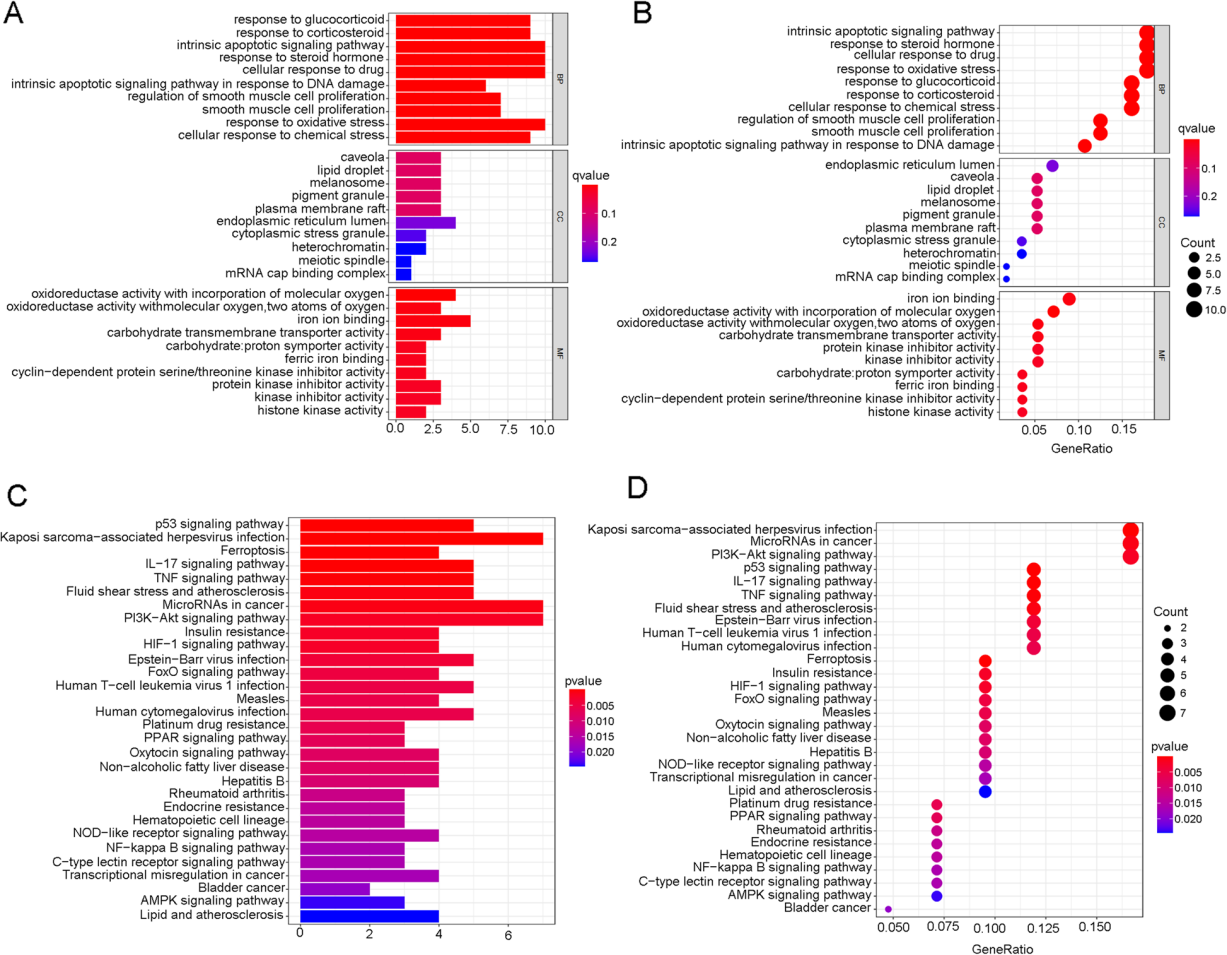
Functional enrichment analysis depending on differentially expressed ferroptosis-related genes between bladder cancer and normal tissues in TCGA cohort. **A** Gene Ontology enrichment analysis by a bar plot. **B** Gene Ontology enrichment analysis by a bubble chart. **C** KEGG enrichment analysis by a bar plot. **D** KEGG enrichment analysis by a bubble chart

**Fig. 2:**
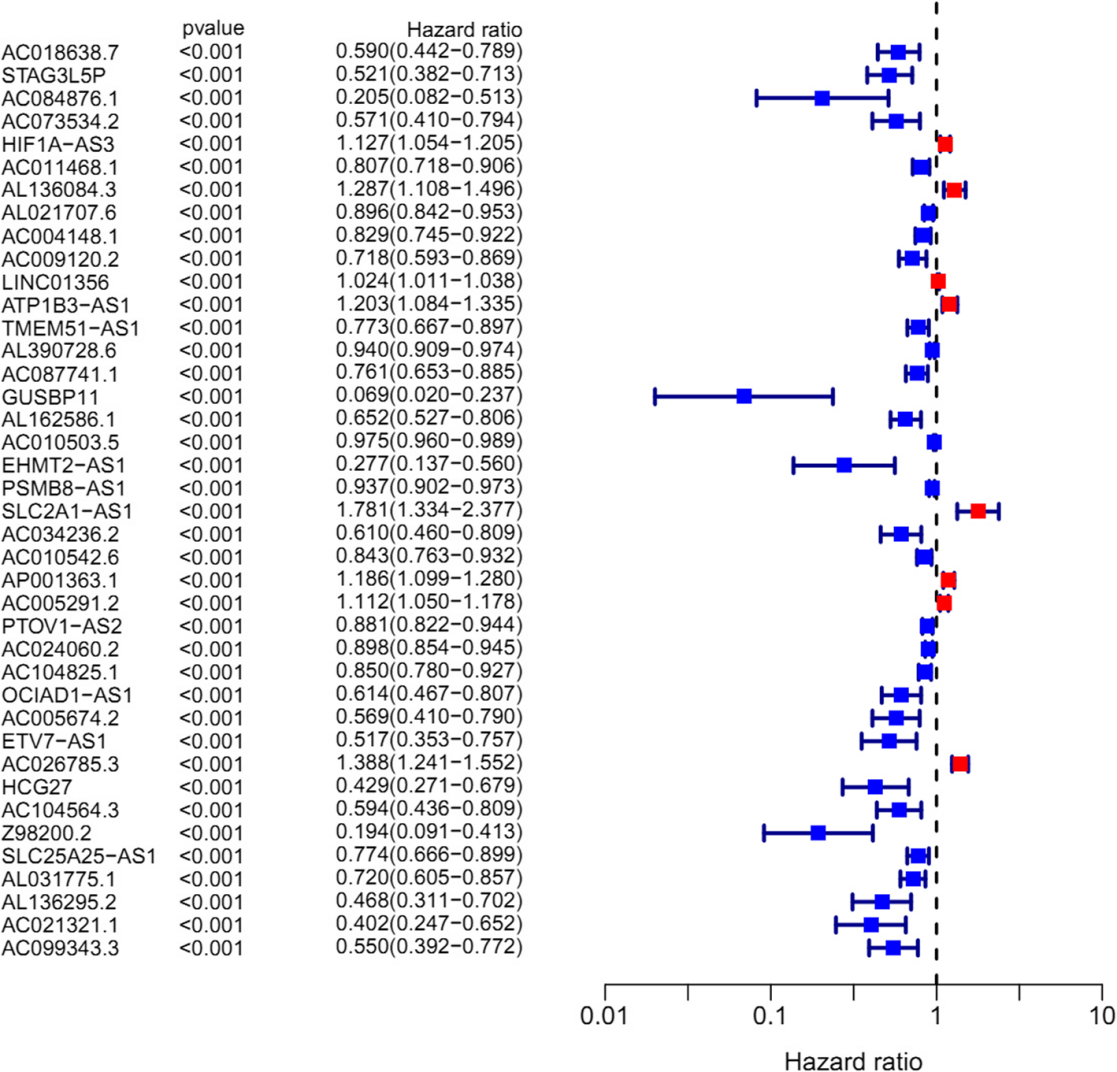
The prognostic ferroptosis lncRNAs during univariate regression analysis in TCGA cohort

A total of 382 ferroptosis-related genes were identified and the detailed genes were cross-referenced by Supplementary Table 10. The detailed expression data of ferroptosis-related genes for the TCGA cohort were then documented in supplementary table 11. Depending on the ferroptosis genes expression and lncRNA expression data from supplementary table 11 and supplementary table 6, Pearson’s correlation analysis was performed to conduct co-expression analysis between ferroptosis mRNA and lncRNA. The co-expression analysis results were shown in supplementary table 12. A total of 1759 lncRNAs were identified during co-expression analysis when defining the |correlation coefficients| > 0.4 and *p* < 0.001. These co-expression lncRNAs with ferroptosis-related genes were named ferroptosis lncRNAs, which were cross-referenced in supplementary table 13. Then, the differentially expressed ferroptosis genes and ferroptosis lncRNAs were further identified in the TCGA cohort when defining |log FC|> 1and FDR < 0.05. A total of 59 ferroptosis genes and 538 ferroptosis lncRNAs were identified (Supplementary Tables 14 and 15). The detailed DEG expression data were cross-referenced in supplementary tables 16 and 17. Furthermore, the GO enrichment results were illustrated by barplot (Fig. 1A) and bubble (Fig. 1B). The biological activities of “response to oxidative stress”, “response to glucocorticoid”, “cellular response to chemical stress”, and “intrinsic apoptotic signalling pathway” were significantly enriched during GO analysis. The KEGG results were also illustrated by barplot (Fig. 1C) and bubble (Fig. 1D). Various mechanism pathways including the “p53 signalling pathway”, “PI3K−Akt signalling pathway”, and “TNF signalling pathway” were significantly enriched during KEGG analysis. The above signal pathways have been also reported to have a close relationship with ferroptosis. Then, the clinical data and lncRNA expression levels were merged into one document (Supplementary Table 18) to identify the prognostic ferroptosis lncRNAs. The univariate Cox analysis identified a total of 40 prognostic ferroptosis lncRNAs. The prognostic analysis results are shown in Fig. 2 and the detailed expression levels of prognostic lncRNAs were cross-referenced in supplementary table 19. Then the prognostic ferroptosis-related lncRNAs were included in the further multivariate Cox regression analysis to evaluate each prognostic contribution for patients’ overall survival. 17 prognostic ferroptosis lncRNAs were used to construct the ferroptosis lncRNA signature depending on the risk score formula in “Materials” section. (Supplementary Table 20) Then the median risk score was used to group TCGA cohorts into high- and low-risk groups. The detailed scoring results of the TCGA cohort were cross-referenced in supplementary table 21. Depending on the same method, the risk scores of the GEO cohort were calculated out and the median score of the GEO cohort was used to group this cohort into high- and low-risk groups. The detailed scoring results of the GEO cohort were also cross-referenced in supplementary table 22.

**Fig. 3:**
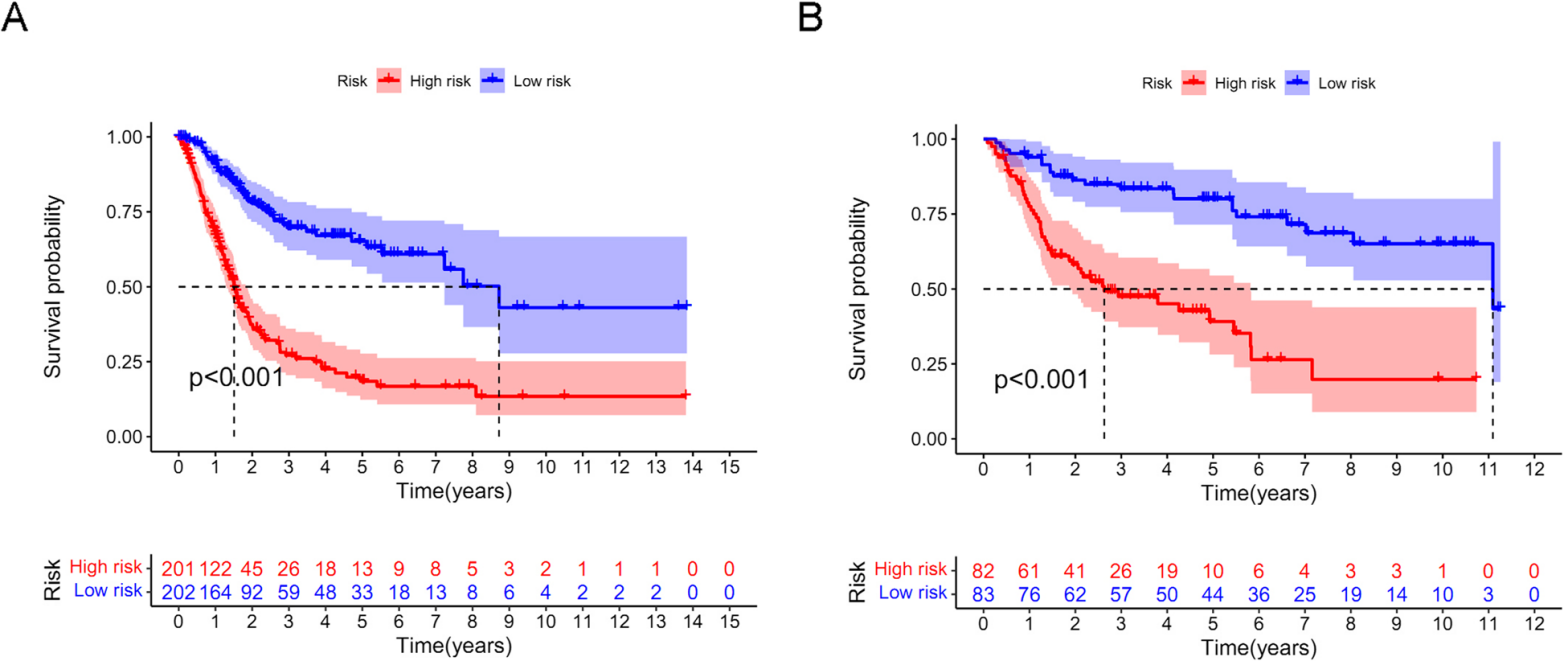
The prognostic value for overall survival of ferroptosis lncRNA risk signature in this study. **A** Kaplan-Meier curve analysis of lncRNA risk signature for overall survival in the TCGA cohort. **B** Kaplan-Meier curve analysis of lncRNA risk signature for overall survival in the GEO cohort

### Validation of lncRNA signatures including Kaplan-Meier analysis, independent prognostic analysis, ROC analysis and decision curve analysis

**Fig. 4:**
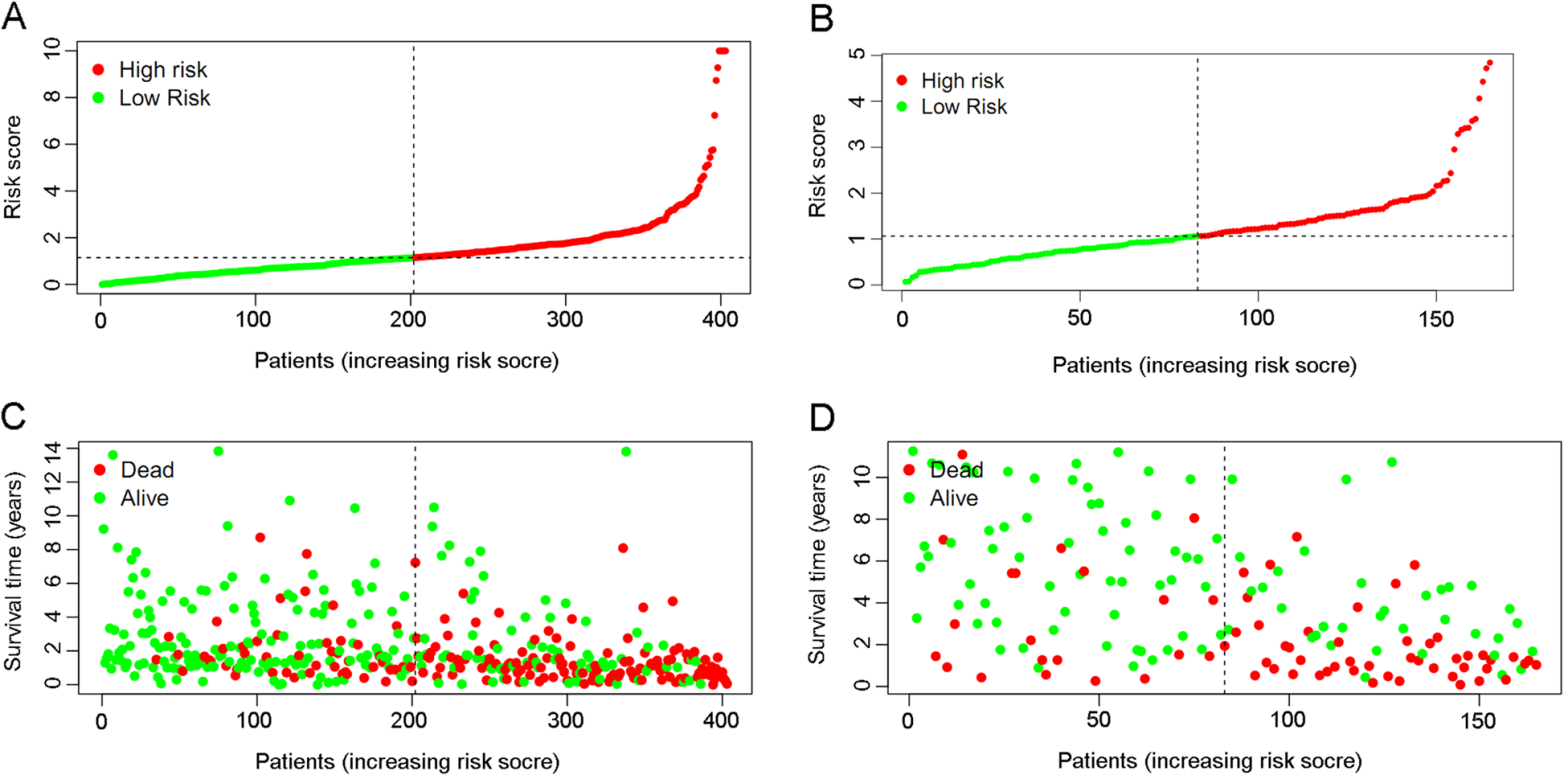
The distribution of lncRNA risk scores and survival status in both TCGA and GEO cohort. **A** The distribution of risk score in TCGA cohort. **B** The distribution of risk score in GEO cohort. **C** The distribution of survival status in TCGA cohort. **D** The distribution of survival status in GEO cohort

**Fig. 5:**
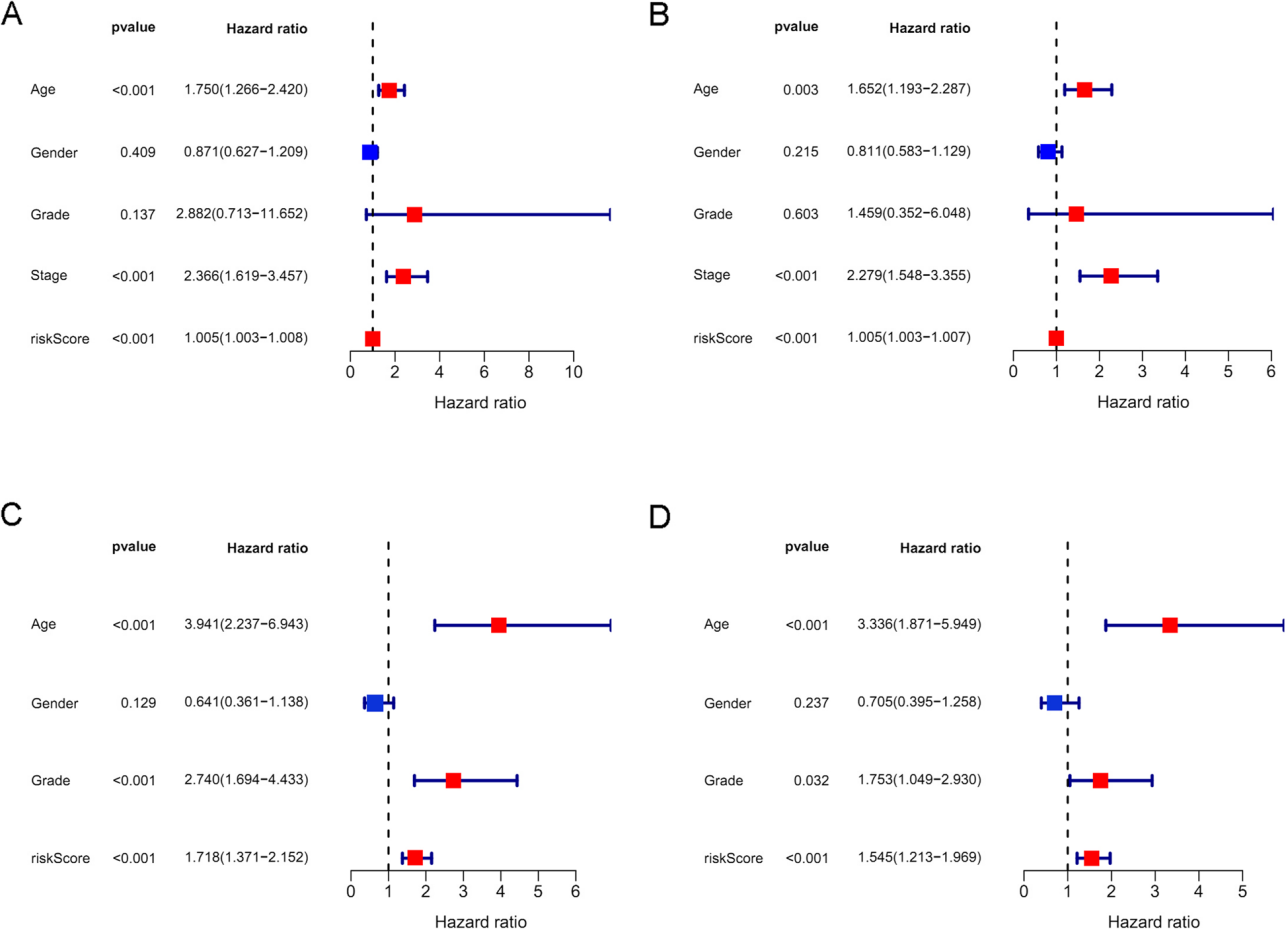
The univariate and multivariate regression analysis in both TCGA and GEO cohorts. **A** The univariate regression analysis results for the TCGA cohort. **B** The multivariate regression analysis results for the TCGA cohort. **C** The univariate regression analysis results for the GEO cohort. **D** The multivariate regression analysis results for the GEO cohort

**Fig. 6:**
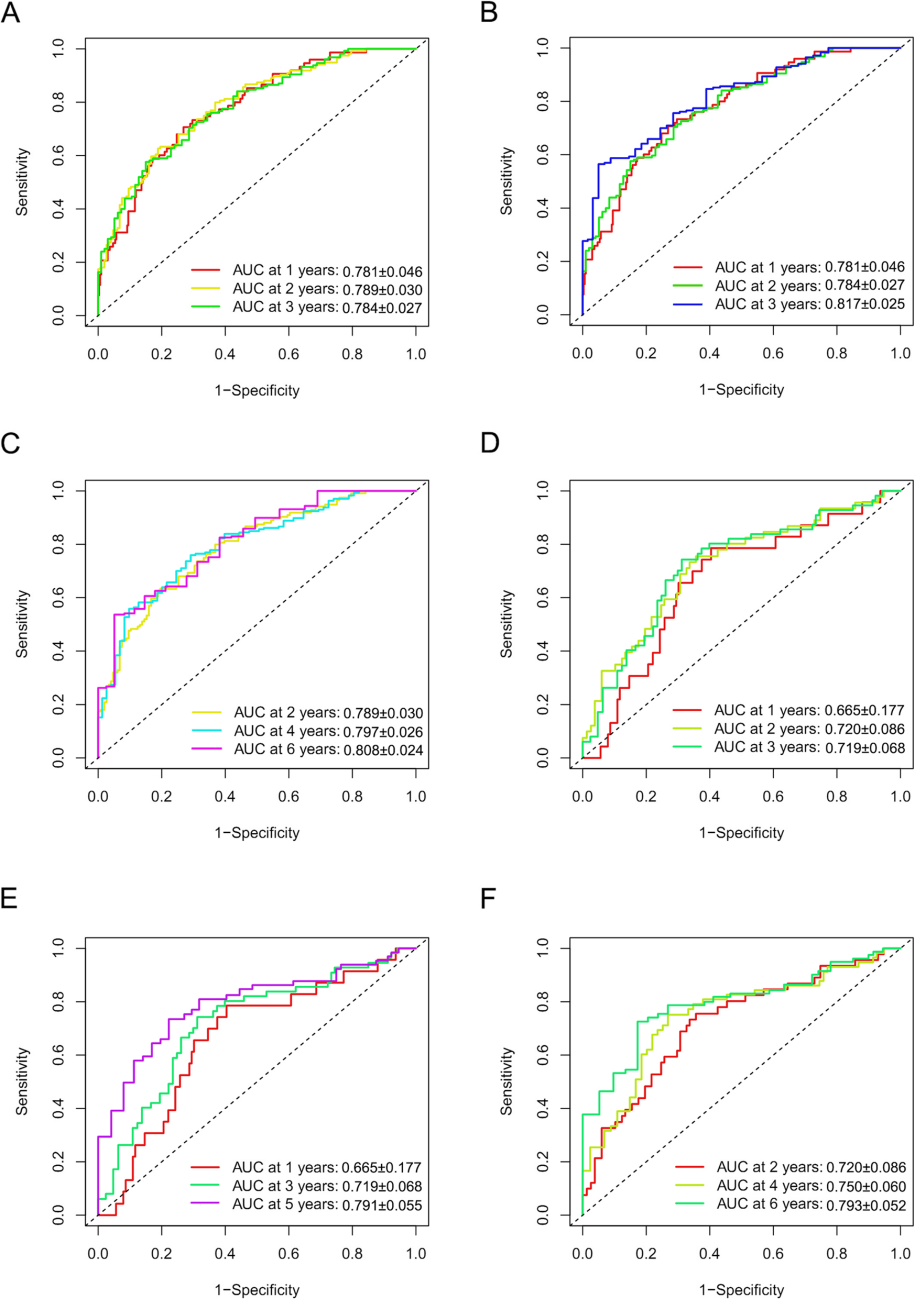
The ROC analysis results of ferroptosis lncRNA risk scores for overall survival outcomes. **A** The ROC curve of risk score for overall survival outcomes stratified by 1, 2, 3 years in the TCGA cohort. **B** The ROC curve of risk score for overall survival outcomes stratified by 1, 3, 5 years in the TCGA cohort. **C** The ROC curve of risk score for overall survival outcomes stratified by 2, 4, 6 years in the TCGA cohort. **D** The ROC curve of risk score for overall survival outcomes stratified by 1, 2, 3 years in the GEO cohort. **E** The ROC curve of risk score for overall survival outcomes stratified by 1, 3, 5 years in the GEO cohort. **F** The ROC curve of risk score for overall survival outcomes stratified by 2, 4, 6 years in the GEO cohort

**Fig. 7:**
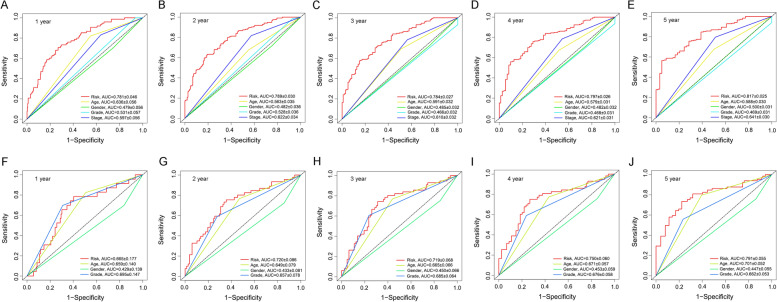
The ROC analysis for various clinical parameters in both TCGA and GEO cohorts. **A** The ROC analysis for various clinical parameters at 1 year in the TCGA cohort. **B** The ROC analysis for various clinical parameters at 2 years in the TCGA cohort. **C** The ROC analysis for various clinical parameters at 3 years in the TCGA cohort. **D** The ROC analysis for various clinical parameters at 4 years in the TCGA cohort. **E** The ROC analysis for various clinical parameters at 5 years in the TCGA cohort. **F** The ROC analysis for various clinical parameters at 1 year in the GEO cohort. **G** The ROC analysis for various clinical parameters at 2 years in the GEO cohort. **H** The ROC analysis for various clinical parameters at 3 years in the GEO cohort. **I** The ROC analysis for various clinical parameters at 4 years in the GEO cohort. **J** The ROC analysis for various clinical parameters at 5 years in the GEO cohort

**Fig. 8:**
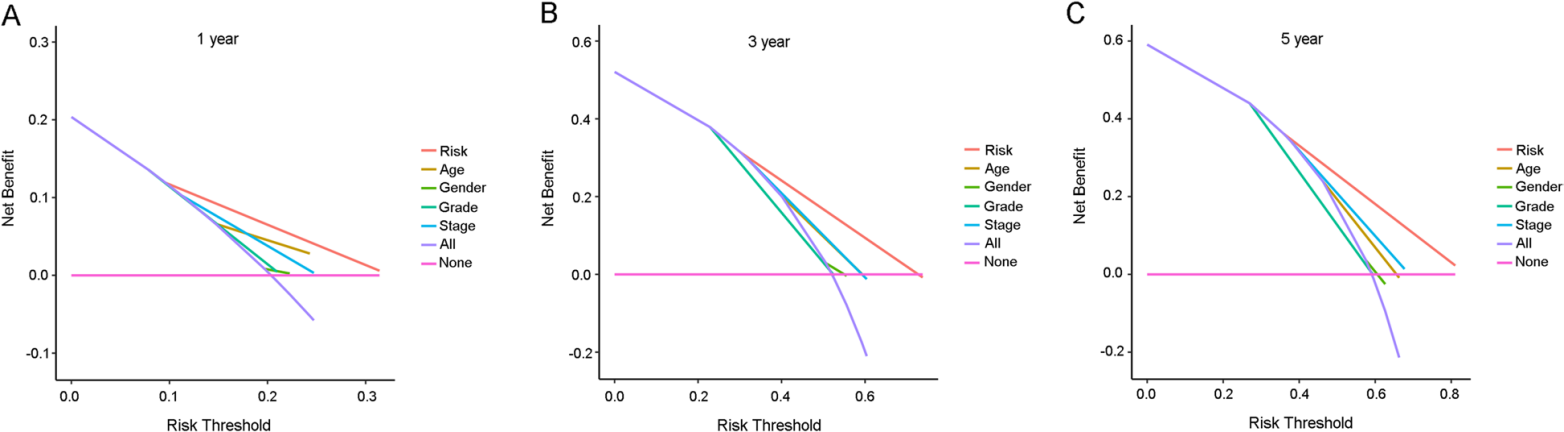
The DCA analysis in the TCGA cohort. **A** The DCA analysis at 1 year time in the TCGA cohort. **B** The DCA analysis at 3 years in the TCGA cohort. **C** The DCA analysis at 5 years in the TCGA cohort

Figure 3 A and B show that the high-risk groups tended to get a significant poorer survival outcomes compared to low-risk groups in both the TCGA cohort (*p* < 0.001) and the GEO cohort (*p* < 0.001). Figure 4 A illustrates the distribution of lncRNA risks cores and Fig. 4 C shows the proportion of patients’ survival status in different risk groups in the TCGA cohort. Figure 4 C shows that the proportion of “dead” status in the high-risk group was significantly higher than that in the low-risk group. Similarly, Fig. 4 B illustrates the distribution of lncRNA risks cores and Fig. 4 D shows the proportion of patients’ survival status in different risk groups in the GEO cohort. Figure 4 D shows that the proportion of “dead” status in the high-risk group was significantly higher than that in the low-risk group for the GEO cohort. Figure 4 intuitively indicates the accurate predictive value of the lncRNA risk group. Depending on the risk grouping results (Supplementary Table 21) and downloaded clinical data (Supplementary Table 2) for the TCGA cohort, Fig. 5 A shows that during univariate regression analysis, the risk score (*p* < 0.001), age (*p* < 0.001), and tumour stage (*p* < 0.001) were significant risk factors for patients’ overall survival. The corresponding multivariate analysis in Fig. 5B further confirmed that the above risk score (*p* < 0.001), age (*p* = 0.003), and tumour stage (*p* < 0.001) were independent risk factors for overall survival in TCGA cohorts. Interestingly, the tumour grade lost the prognostic value for bladder cancer during both univariate and multivariate regression analyses when including risk score and other clinical data during this study. Depending on the risk grouping results (Supplementary Table 22) and clinical data (Supplementary Table 4) in the GEO cohort, univariate prognostic analysis in Fig. 5C identified risk score (*p* < 0.001), age (*p* < 0.001), and grade (*p* < 0.001) to be significant risk factors for overall survival. Further multivariate prognostic analysis in Fig. 5D showed that risk score (*p* < 0.001), age (*p* < 0.001) and grade (*p* = 0.032) were independent risk factors for overall survival in the GEO cohort. The ROC analysis results were also illustrated in Fig. 6. Figure6 A shows the ROC analysis results of ferroptosis lncRNA risk score for 1, 2, and 3 years in the TCGA cohort. The AUCs reached to 0.781 ± 0.046, 0.789 ± 0.030, and 0.784 ± 0.027 respectively. Figure 6B shows the ROC analysis of risk score for 1, 3, and 5 years in the TCGA cohort. The AUCs during ROC analysis of 5 years reached 0.817 ± 0.025, which indicated a good predictive value for bladder cancer overall survival. Figure 6C shows the ROC analysis of risk score for 2, 4, and 6 years in the TCGA cohort. The AUCs of ROC analysis were 0.789 ± 0.030, 0.797 ± 0.026 and 0.808 ± 0.024 respectively. The above results indicated a good predictive value of the ferroptosis lncRNA risk scores during the 6-year follow-ups. Similarly, depending on risk grouping results and clinical data (Supplementary Table 22), Fig. 6D shows in the GEO cohort the AUCs of ROC analysis for 1, 2, and 3 years were 0.665 ± 0.177, 0.720 ± 0.086, and 0.719 ± 0.068 respectively. Figure 6E shows that the AUCs of 1, 3, and 5 years were 0.665 ± 0.177, 0.719 ± 0.068, and 0.791 ± 0.055 respectively. Figure 6F shows that AUCs of 2, 4, and 6 years were 0.720 ± 0.086, 0.750 ± 0.060, and 0.793 ± 0.052 respectively. To further confirm the predictive role of risk score in this study, the risk score’s predictive value was also compared with other clinical parameters. Figure 7A–E show AUCs of risk score, age, gender, grade, and stage at 1, 2, 3, 4, and 5 years respectively for the TCGA cohort. From 1 to 5 years, the AUCs of risk score were all significantly higher than other clinical data. The AUCs of stage in TCGA cohort for 1, 2, 3, 4, and 5 years were 0.597 ± 0.056, 0.622 ± 0.034, 0.610 ± 0.032, 0.621 ± 0.031, and 0.641 ± 0.030, which were significantly higher than that of grade. These results were consistent with previous independent risk analysis which indicated the stage, not the grade, was an independent risk factor for bladder cancer. Figure 7F–J show the AUCs of various clinical parameters during 1, 2, 3, 4, and 5 years for the GEO cohort. Figure 7 F shows that the AUC of grade was 0.695 ± 0.140 and AUC of risk was 0.665 ± 0.177, which indicated that the grade posed a better AUC value for predicting 1-year or short-term survival outcomes. However, the AUCs of risk score during 2–5 years were significantly higher than those of grade. The AUC of age during the 5th year was also higher than that of grade, which results further indicated that the grade might play important roles during short-term prognosis and risk and age played important roles during long-term prognosis. Table 1 intuitively illustrates different AUCs for 1–5 years in both TCGA and GEO cohorts. The DCA analysis was also performed in the TCGA cohort to explore the prognostic role of risk score in this study. Figure 8A–C show the DCA analysis of 1, 3, and 5 years of various clinical parameters in the TCGA cohort. Three figures intuitively showed that the risk score posed the best performance during DCA curves of both 1, 3, and 5 years.

### Co-expression network illustration and function enrichment analysis

**Fig. 9:**
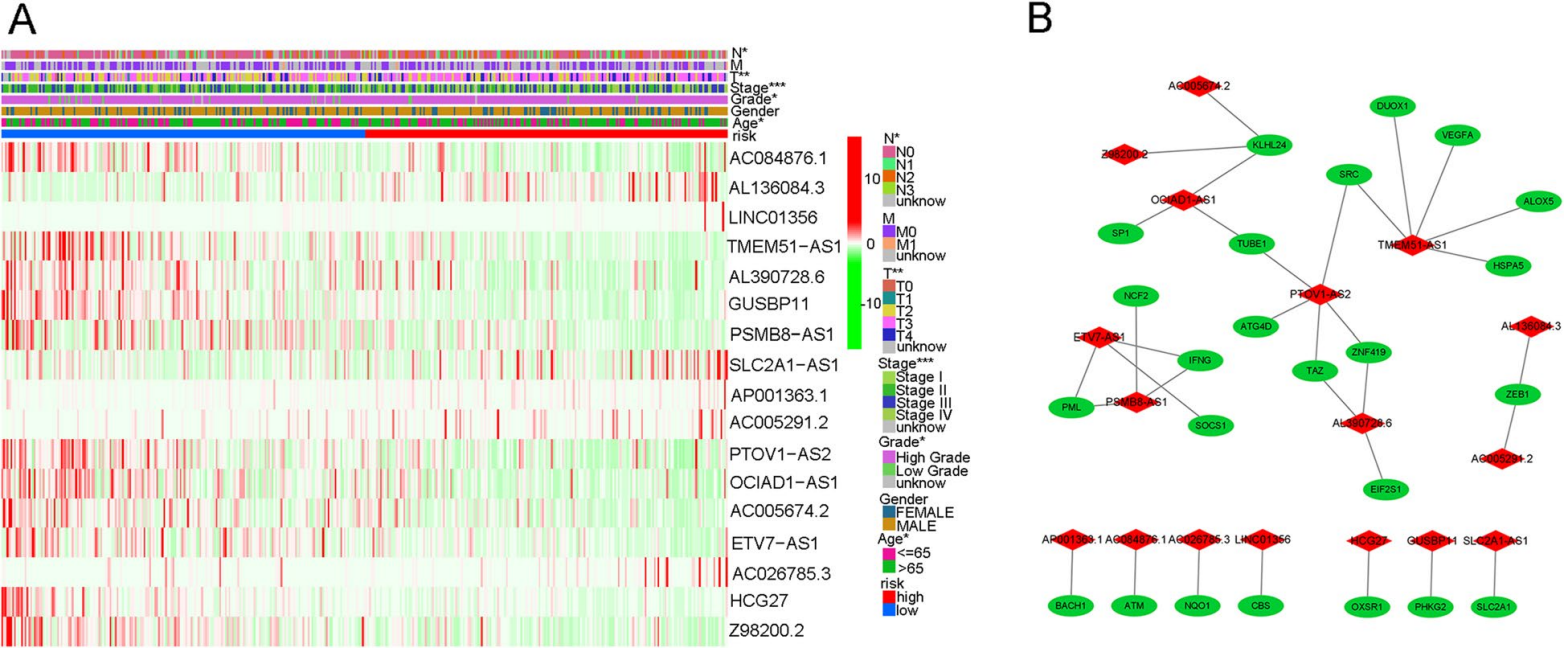
The heatmap and co-expression network depending on ferroptosis lncRNAs in TCGA cohort. **A** The heatmap illustrating the relationships between lncRNA risk scores and various clinical parameters. **B** The co-expression networks between ferroptosis lncRNAs and mRNAs. The asterisk symbol indicates the statistical *p*-value. (*p<0.05; **p<0.01; ***p<0.001). The red colour presents lncRNAs and green colour presents mRNAs

**Fig. 10:**
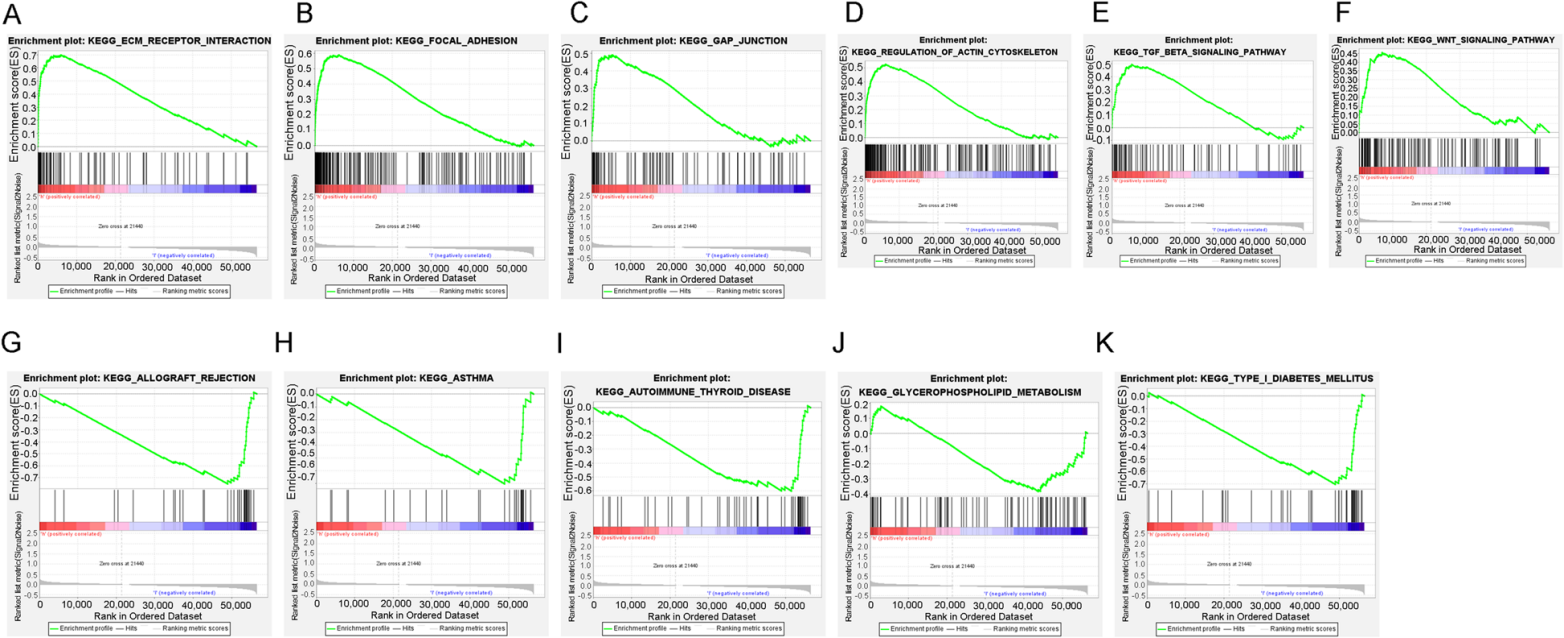
The GSEA enrichment results depending on DEGs between high- and low-risk groups in the TCGA cohort. **A**–**F** Significantly enriched biological activities in high-risk groups. **G**–**K** Significantly enriched biological activities in the low-risk group

Figure 9 A shows that the stage (*p* < 0.001), grade (*p* < 0.05), and age (*p* < 0.05) posed a significant correlation with risk groups. The co-expression network was also illustrated in Fig. 9B. The red presented the ferroptosis lncRNAs during signature and the green presented the co-expression mRNAs in this study. Depending on the risk grouping results (Supplementary Table 21) and whole transcriptome expression file (Supplementary Table 1), functional enrichment analysis using GSEA (version 4.1.0) was performed among different risk groups. The top 6 significantly enriched biological activities were illustrated in the high-risk group (Supplementary Table 23) and low-risk group (Supplementary Table 24). (*p* < 0.05) Fig. 10A–F illustrate that the top 6 enriched activities in the high-risk group were regulation of actin cytoskeleton, TGF beta signal pathway, focal adhesion, ECM receptor interaction, GAP junction, and WNT signalling pathway. Figure 10G–K illustrate that the top enriched activities in the low-risk group were asthma, type 1 diabetes mellitus, allograft rejection, glycerophospholipid metabolism, and autoimmune thyroid disease.

### Potential application of ferroptosis lncRNA signature for predicting tumour-immune microenvironment and immunotherapy responses

**Fig. 11:**
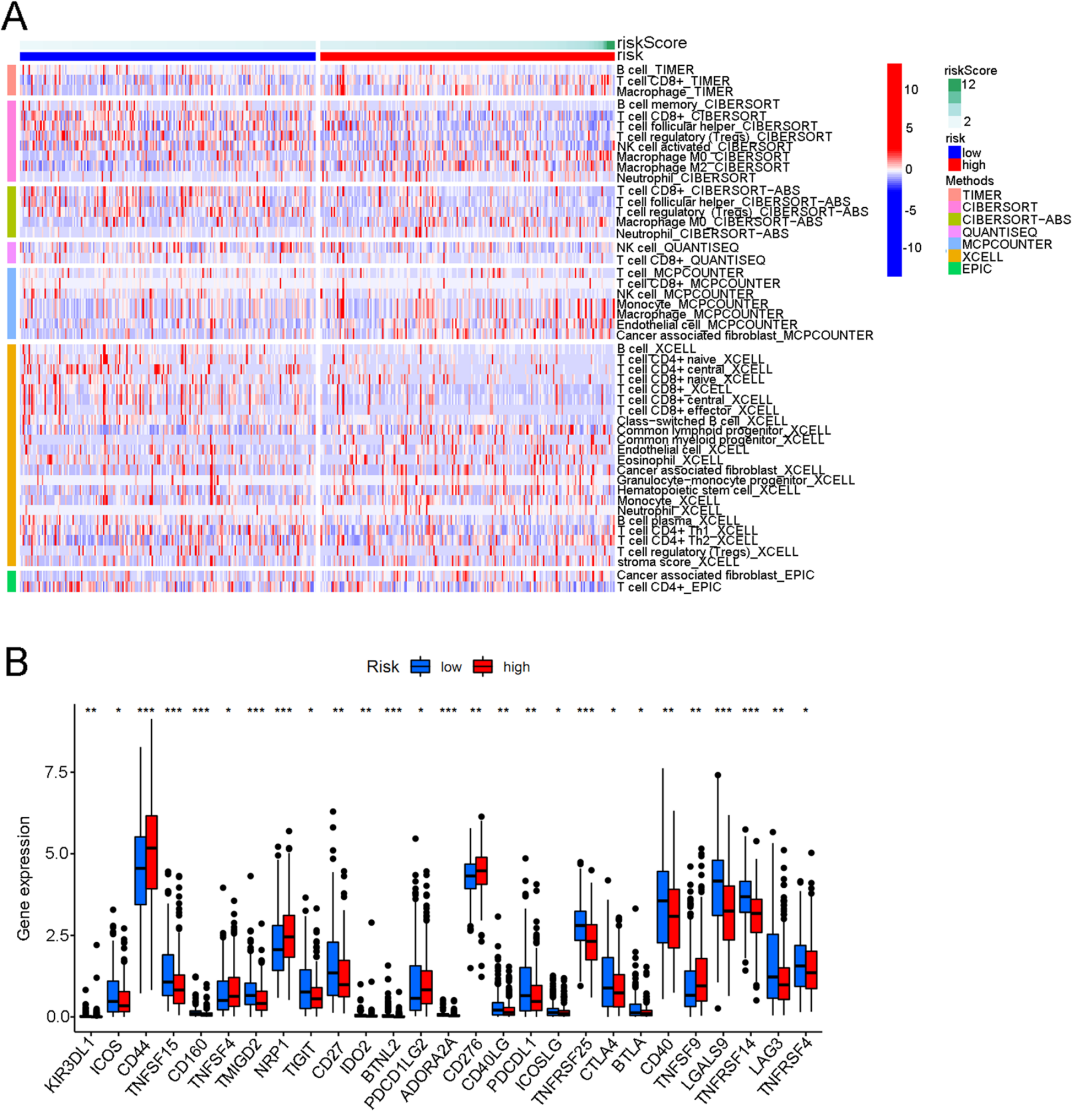
The different infiltration levels of various immune cells and immune checkpoint-related genes expression between high- and low-risk groups in TCGA cohort. **A** The different infiltration levels of various immune cells between high- and low-risk groups. **B** The expression difference of immune checkpoint-related genes between high- and low-risk groups. The asterisk symbol indicates the statistical *p*-value (*p < 0.05; **p < 0.01; ***p < 0.001)

**Fig. 12:**
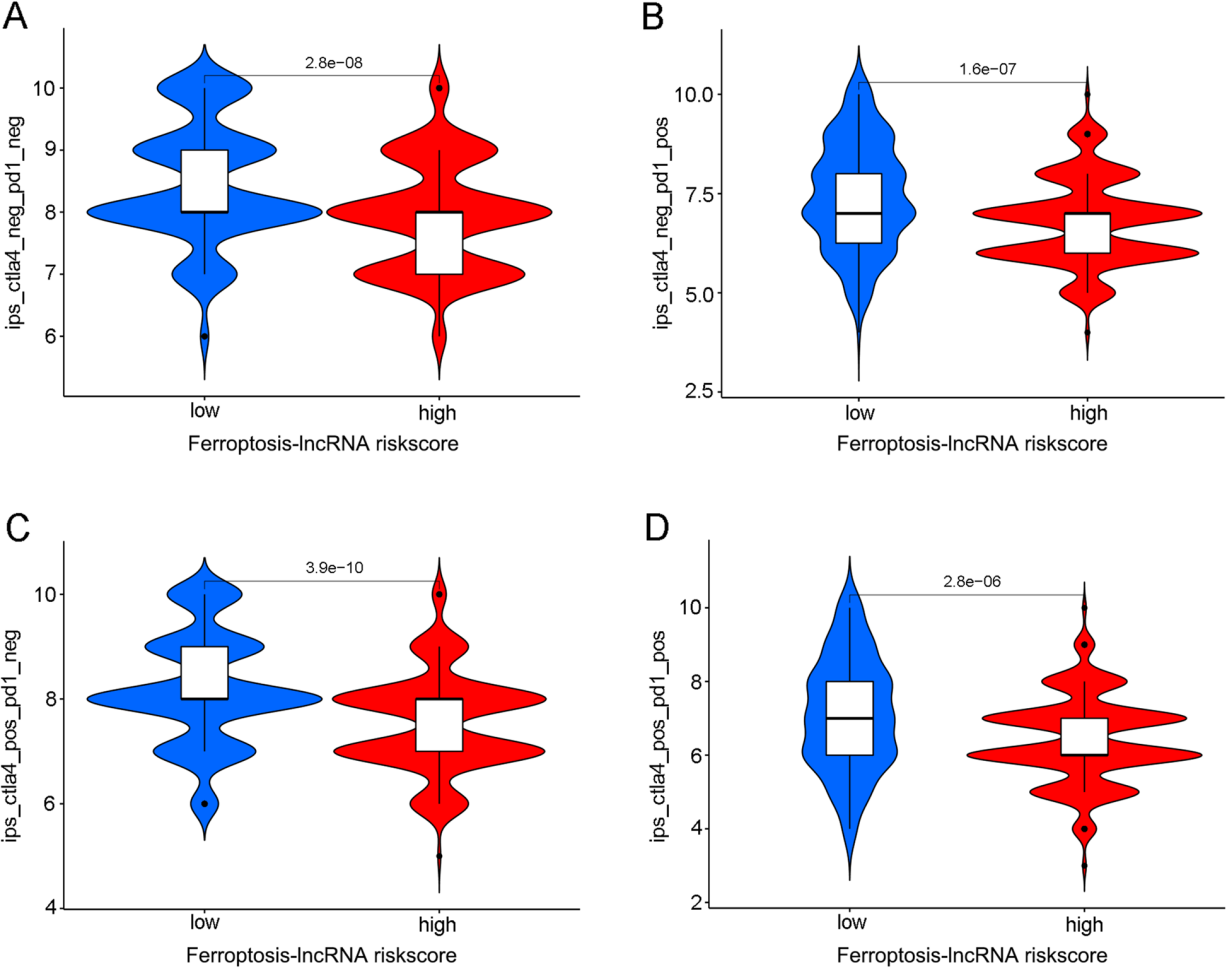
The prediction value of lncRNA risk groups for immunotherapy responses in TCGA cohort. **A** The high-risk group obtained significantly poorer immunotherapy response if no immunotherapy was performed (*p* < 0.001). **B** The high-risk group obtained significantly poorer immunotherapy response if only anti-PD1 immunotherapy was conducted (*p* < 0.001). **C** The high-risk group obtained significantly poorer immunotherapy response if only anti-CTLA4 immunotherapy was used (*p* < 0.001). **D** The high-risk group obtained significantly poorer immunotherapy response even though anti-PD1 and anti-CTLA4 immunotherapy methods were simultaneously conducted (*p* < 0.001)

Figure 11A shows that the infiltration levels of Macrophage_TIMER (*p* < 0.001), Neutrophil_CIBERSORT−ABS (*p* = 0.004), Cancer-associated fibroblast_EPIC (*p* < 0.001), and Macrophage M2_CIBERSORT (*p* < 0.001) were significantly higher in high lncRNA risk groups. The infiltration levels of T cell follicular helper_CIBERSORT, (*p* < 0.001) T cell CD8+_CIBERSORT−ABS, (*p* = 0.003) NK cell activated_CIBERSORT, (*p* = 0.014) T cell CD4+ naive_XCELL (*p* < 0.001) and T cell CD4+ central memory_XCELL (*p* < 0.001) were significantly higher in low lncRNA risk groups. Figure 11 B illustrates that all reported immune checkpoint genes’ expressions were significantly different in high- and low-risk groups. The expression levels of PD1, PD-L1, and CTLA4 were significantly downregulated in the high-risk group. (*p* < 0.05) The predictive value for immunotherapy response of the ferroptosis lncRNA risk groups was also explored using the immunotherapeutic cohort from http://tcia.at/. For all immunotherapy strategies, the high lncRNA risk group tended to get a significantly poorer response than the low-risk group (Fig. 12).

## Discussion

Bladder cancer was reported to be the most common urothelial carcinoma, and its increasing incidence had posed a great threat to human health [32]. He et al. reported that overall incidence of bladder cancer increased over past decades and various clinical parameters including age at diagnosis, differentiated grade, regional lymph nodes removed status and tumour size were independent risks for cancer prognosis [33]. With the development of sequencing technology, increasing researches had pointed out that genetic expressions could be utilized to predict prognosis of various malignancies [34]. Our previous study had confirmed that ferroptosis-related genes played important roles in the development of bladder cancer [9]. This study continues to explore whether ferroptosis-related lncRNAs play any role in bladder cancer. We further explored the possible predictive role of lncRNAs by assessing infiltrating levels of immune cells in the tumour microenvironment and immunotherapy responses in bladder cancers. This study, along with our previous papers, further revealed the future treatment targets and potential biomarkers of bladder cancer. The ferroptosis-related lncRNAs in our study exhibited a good predictive capacity for overall survival in bladder cancer. Additionally, they could be utilized to reveal tumour immune microenvironment and immunotherapy responses. Recent study also constructed a lncRNA pair model with the exact expression which could predict the prognosis of bladder cancer patients [35]. Thus, investigating lncRNA roles for bladder cancer could further discover potential molecular mechanism for cancer development and therapy.

This study showed that the biological activities of “response to oxidative stress”, “response to glucocorticoid”, “cellular response to chemical stress” and “intrinsic apoptotic signalling pathway” were significantly enriched during bladder cancer depending on ferroptosis related genes. These results indicated potential relationships between ferroptosis and apoptotic pathways as well as various metabolic pathways. The KEGG analysis also identified a close relationship between the p53 signalling pathway. The p53 acted as a tumour suppressor factor by taking part in cell survival regulation procedures. Previous studies also reported that p53 could regulate ferroptosis from different mechanisms [36]. Firstly, p53 could promote ferroptosis through inhibiting the expression levels of solute carrier family 7 member 11. Secondly, p53 could also inhibit ferroptosis by suppressing the dipeptidyl peptidase 4 activity or by upregulating the expression of cyclin-dependent kinase inhibitor 1A. Our study results also indicated a role of p53 for ferroptosis during bladder cancer.

Depending on the ferroptosis lncRNAs, the high-risk group tended to get significantly poorer survival outcomes than low-risk groups, both in the TCGA cohort and the GEO cohort. Furthermore, Fig. 4 A and C indicate a higher proportion of dead patients for the high-risk group in the TCGA cohort. Figure 4 B and D also indicate a higher proportion of dead patients for the high-risk group. All the above results confirmed the prognosis predictive role of the risk group depending on ferroptosis lncRNAs in bladder cancer. For the TCGA cohort, risk score, age, and tumour stage were identified as independent risk factors for overall survival. For the GEO cohort, the risk score, age, and grade were identified as independent risk factors for overall survival. Interestingly, the tumour grade lost the prognostic value for bladder cancer during both univariate and multivariate regression analyses when including risk score, tumour stages, and other clinical data in the TCGA cohort. However, the tumour grade was indeed identified as independent risk factors for GEO cohort, during which cohort the tumour stage information was unavailable during analysis. We thought the above different results were caused by the high linear relationship between clinical stages and pathological grades during multivariate regression analysis [37]. The low grade usually presented as a low progression characteristic and usually got an earlier clinical stage. Similarly, the tumours with high grade also tended to get an advanced clinical stage evaluation. However, in both the GEO cohort and the TCGA cohort, the ferroptosis lncRNA risk scores were both identified as independent risk factors for survival outcomes. This result further confirmed the predictive value for survival outcomes of our lncRNA risk scores.

The ROC analysis showed that the ferroptosis lncRNA risk signature posed accurate prediction value for overall survival in both TCGA cohort and GEO cohort. The AUCs of risk score gradually increased from 1 year to 5 years in the TCGA cohort. This result indicated a good prediction role for patients’ long-term prognosis in the TCGA cohort. Furthermore, in the GEO cohort, the AUC of 1 year was only 0.665 ± 0.177. The results during GEO also confirmed that the ferroptosis lncRNA risk score could be used as a valuable predictive tool for long-term prognosis. The short-term prognosis might be significantly influenced by various perioperative elements including operation method, tumour’s anatomical complexity, and perioperative complications. The ferroptosis lncRNA risk score in our study could indicate overall survival outcomes from the perspective of genetic mechanism, which was theoretically more accurate than various clinical or conventional parameters. Different AUCs of various clinical parameters during 1–5 years were also compared. In the TCGA cohort, The AUCs of ferroptosis lncRNA risk scores were significantly greater than other parameters including age, clinical stage, and tumour grade during 1–5 years. Interestingly, the AUCs of the clinical stage were all greater than those of grade, which results were consistent with our above multivariate regression analysis in the TCGA cohort. In the GEO cohort, the grade posed a greater AUC than our risk score at 1 year time. However, the AUCs of grade were gradually overtaken by risk score and age over time. Perhaps, the advanced grade might increase tumour’s anatomical complexity and elevate surgical difficulty, which further significantly affected short-term prognosis. The risk score and age posed a good predictive role for long-term prognosis in ROC analysis in the GEO cohort. The following DCA analysis results also confirmed the accurate prediction value of our ferroptosis lncRNA risk score. A significant relationship between clinical stage, grade, and patients’ ages was also indicated in this study. With the rapid development of gene sequencing technology, our ferroptosis lncRNA risk score or our previous ferroptosis mRNA risk score would become a valuable prediction tool for overall survival in bladder cancer patients.

Further functional enrichment analysis found the top 6 enriched activities in the high-risk group were regulation of actin cytoskeleton, TGF beta signalling pathway, focal adhesion, ECM receptor interaction, GAP junction, and WNT signalling pathway. On the other hand, the top enriched activities in the low-risk group included various abnormal immunity activities including asthma, type 1 diabetes mellitus, allograft rejection, glycerophospholipid metabolism, and autoimmune thyroid disease. Our results were consistent with previous studies which also reported a close relationship between ferroptosis and TGF beta signal pathway, focal adhesion, ECM receptor interaction, GAP junction, and WNT signalling pathway [38, 39]. Various metabolic activities correlated with ferroptosis inhibition and further promoted malignant tumour development. This study result implicated a possible treatment direction for the regulation of ferroptosis induction or inhibition in the future. The low-risk groups obtained enrichment results of various abnormal immunity activities. The allograft rejection, autoimmune thyroid disease, and asthma all presented as an overactive station of the immune system. Furthermore, this study showed that the infiltration levels of Macrophage, Macrophage M2, which correlated with immune suppression, were significantly higher in high lncRNA risk groups. The infiltration levels of T cell follicular helper_CIBERSORT, T cell CD8+_CIBERSORT−ABS, NK cell activated_CIBERSORT, T cell CD4+ naive_XCELL, and T cell CD4+ central memory_XCELL were significantly higher in low lncRNA risk groups. The correlation between immune activities and ferroptosis had been unclear during the past years. On one hand, some researchers reported that immune activity might promote or induce ferroptosis through system xc-downregulation mechanism [40]. On the other hand, the occurrence of ferroptosis could produce many metabolites which could in turn upregulate the immune system activities about the presence of ferroptosis-sensitive cells [41]. Considering our analysis results, we supposed that the ferroptosis overactive station in the low-risk group led to a good survival prognosis and the ferrptosis-related products also activated the immune system which was responsible for various autoimmune diseases in our study. The deeper researches on this topic might provide potential treatment direction for bladder cancer in the future.

The relationships between the risk groups and genes corresponding to various immune checkpoints as well as m6A-related genes were explored in this study. Most m6A-related genes including *ZC3H13*, *WTAP*, *FTO*, *METTL3,* and *YTHDC1* exhibited significantly different expression levels between high and low-risk groups. This result was consistent with various previous studies which also identified that m6A-related genes could significantly promote the development of bladder cancer [42, 43]. A total of 27 immune checkpoint–related genes were included in this study. Almost all such genes exhibited significant relationships with the risk groups (*p* < 0.05). The expression levels of *PD1*, *PD-L1,* and *CTLA4* were significantly lower in the high-risk group than in the low-risk group. Low expression levels of *PD1*, *PD-L1,* and *CTLA4* were reported to be responsible for poor immunotherapy responses in various malignant cancers [40]. Furthermore, this study showed that the high-risk group in this study tended to have poorer immunotherapy responses than the low-risk group, irrespective of the applied immunotherapy strategy. The poor immunotherapy responses in the high-risk group might be caused by the low expression of genes related to immune checkpoints in bladder cancer. All these results indicated that ferroptosis-related lncRNA risk score could be used to predict immune microenvironment and immunotherapy response in the future.

There were also several limitations in this study. Firstly, our lncRNA risk signature was derived from public datasets including both TCGA and GEO cohorts, more external cohorts with transcriptome sequencing information were needed to investigate lncRNA roles in bladder cancer. Secondly, the molecular mechanism of ferroptosis lncRNAs included in our signature was also uncertain and further basic experiments were urgently needed to discover detailed pathways. Furthermore, the ratio between tumour samples and normal samples was 411 vs 19 in the TCGA cohort and 188 vs 68 in the GEO cohort. The unbalanced ratio in this study potentially affected the accuracy of DEG analysis and functional enrichment analysis. Larger cohorts including more normal samples in multi-centres were further needed to validate this study. It was worth mentioning that only tumour samples with available clinical information were included in univariate and multivariate analyses to construct the ferroptosis lncRNA risk signature, thus the limited number of normal samples did not affect the accuracy and reliability of our lncRNA risk signature. Our study demonstrated that ferroptosis lncRNA signature could be utilized to accurately predict survival prognosis and immunotherapy responses in bladder cancer.

## Conclusions

Ferroptosis-related lncRNAs might play vital roles in bladder cancer development. The lncRNA signature could be used to accurately predict survival prognosis and immunotherapy responses in bladder cancer. Ferroptosis-related lncRNAs could serve as a novel therapeutic target in the future.

## Supplementary Information

**Additional file 1: Figure S1.** Most m6A-related genes including ZC3H13, WTAP, FTO, METTL3, and YTHDC1 exhibited significantly different expression levels between high and low-risk groups. (*p<0.05; **p<0.01; ***p<0.001).

**Additional file 2: Table S1.** The expression matrix of TCGA cohort. **Table S2.** The downloaded clinical files of TCGA cohort.** Table S3.** The expression matrix of GEO cohort. **Table S4.** The downloaded clinical files of GEO cohort. **Table S5.** The Genome Reference file discriminating lncRNAs and mRNAs. **Table S6.** The expression matrix of lncRNAs during TCGA cohort. **Table S7.** The expression matrix of mRNAs during TCGA cohort. **Table S8.** The expression matrix of lncRNAs during GEO cohort. **Table S9.** The expression matrix of mRNAs during GEO cohort. **Table S10.** The detailed list of ferroptosis-related genes. **Table S11.** The expression matrix of ferroptosis-related genes during TCGA cohort. **Table S12.** The co-expression analysis results between lncRNAs and mRNAs. **Table S13.** The expression matrix of ferroptosis lncRNAs. **Table S14.** Detailed list of 59 differentially expressed ferroptosis genes between bladder cancer and normal tissues. **Table S15.** Detailed list of 538 differentially expressed ferroptosis lncRNAs between bladder cancer and normal tissues. **Table S16.** Detailed expression matrix of differentially expressed ferroptosis genes between bladder cancer and normal tissues. **Table S17.** Detailed expression matrix of differentially expressed ferroptosis lncRNAs between bladder cancer and normal tissues. **Table S18.** The merged document including both ferroptosis lncRNA expression and clinical information. **Table S19.** Detailed expression matrix of prognostic ferroptosis lncRNAs. **Table S20.** Detailed ferroptosis lncRNAs included in risk signature. **Table S21.** Detailed risk results depending on lncRNA risk signature in TCGA cohort. **Table S22.** Detailed risk results depending on lncRNA risk signature in GEO cohort. **Table S23.** The significantly enriched biological activities during high risk group. **Table S24.** The significantly enriched biological activities during low risk group. **Table 25.** The infiltration levels of various immune cells from http://timer.comp-genomics.org. **Table S26.** The detailed list of immune checkpoints-related genes. **Table S27.** The immunotherapy scoring information for TCGA cohort.

## Data Availability

All data analysed in this study were from public database of TCGA (https://portal.gdc.cancer.gov/), GEO (https://www.ncbi.nlm.nih.gov) and TCIA (http://tcia.at/). All procedure in this study followed corresponding guidelines and relative policies of above public database.

## Abbreviations

TCGA: The Cancer Genome Atlas
GEO: Gene Expression Omnibus
KEGG: Kyoto Encyclopedia of Genes and Genomes
m6A: N6-Methyladenosine modification
GO: Gene Ontology
PD-1: Immune checkpoint programmed cell death 1
ssGSEA: Single-sample gene set enrichment analysis
PD-L1: Immune checkpoint programmed cell death 1 ligand
DEGs: Differentially expressed genes
CTLA-4: Cytotoxic T lymphocyte antigen 4
RB1: Retinoblastoma 1
GSVA: Gene set variation analysis
ROC: Receiver operating characteristic
AUC: Area under curves
DCA: Decision curve analysis
