# Supplementary figures and images for: Predictive role of ferroptosis-related long non-coding RNAs in bladder cancer and their association with immune microenvironment and immunotherapy response
Source: World J Surg Oncol. 2022 Feb 24;20:47. doi: 10.1186/s12957-022-02514-4 (PMC8867683; doi:10.1186/s12957-022-02514-4)

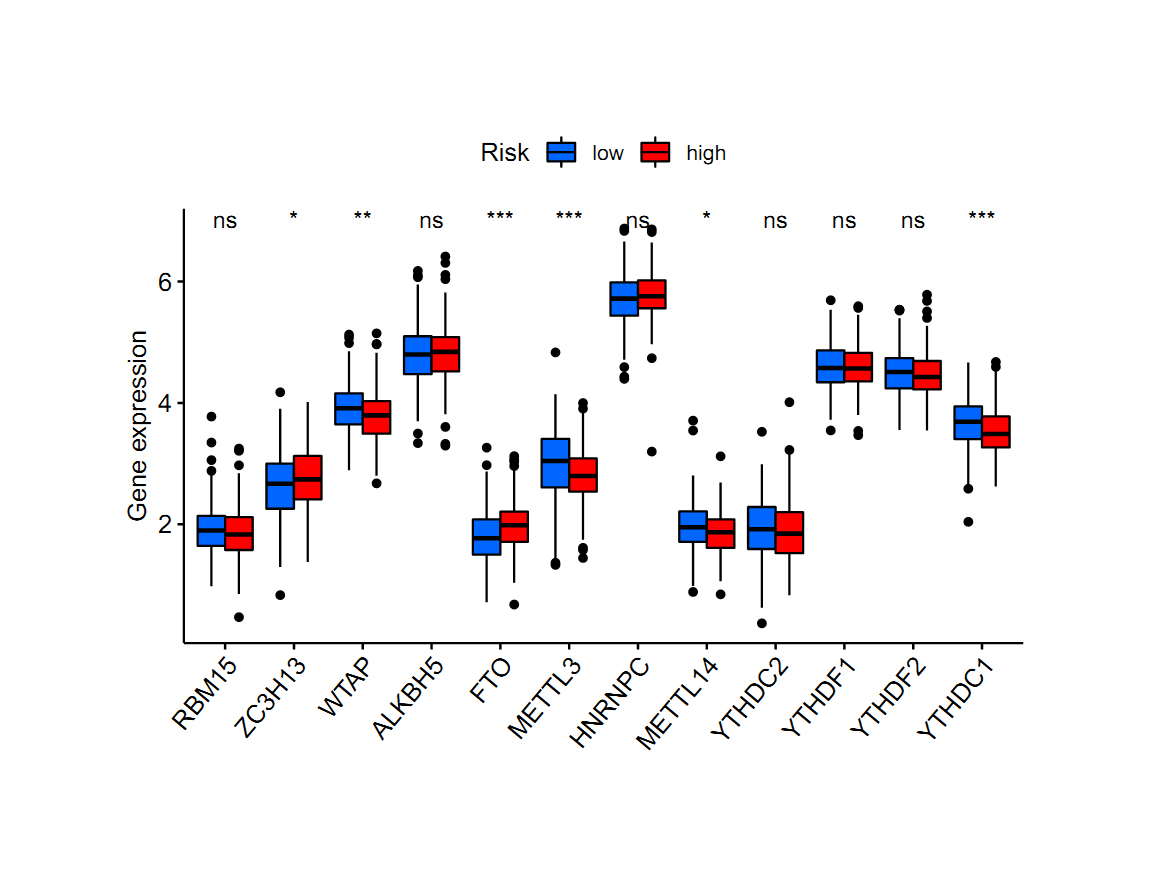

Supplement: Supplementary file 1 — Additional file 1: Figure S1. Most m6A-related genes including ZC3H13, WTAP, FTO, METTL3, and YTHDC1 exhibited significantly different expression levels between high and low-risk groups. (*p<0.05; **p<0.01; ***p<0.001). [file 12957_2022_2514_MOESM1_ESM.jpg]
